# Benefits of a Ball and Chain: Simple Environmental Enrichments Improve Welfare and Reproductive Success in Farmed American Mink (*Neovison vison*)

**DOI:** 10.1371/journal.pone.0110589

**Published:** 2014-11-11

**Authors:** Rebecca K. Meagher, Jamie Ahloy Dallaire, Dana L. M. Campbell, Misha Ross, Steen H. Møller, Steffen W. Hansen, María Díez-León, Rupert Palme, Georgia J. Mason

**Affiliations:** 1 Department of Animal and Poultry Science, University of Guelph, Guelph, Ontario, Canada; 2 Animal Welfare Program, Faculty of Land and Food Systems, University of British Columbia, Vancouver, British Columbia, Canada; 3 Department of Animal Science, Aarhus University, Aarhus, Denmark; 4 Department of Biomedical Sciences/Biochemistry, University of Veterinary Medicine, Vienna, Austria; CNRS, France

## Abstract

Can simple enrichments enhance caged mink welfare? Pilot data from 756 sub-adults spanning three colour-types (strains) identified potentially practical enrichments, and suggested beneficial effects on temperament and fur-chewing. Our main experiment started with 2032 Black mink on three farms: from each of 508 families, one juvenile male-female pair was enriched (E) with two balls and a hanging plastic chain or length of hose, while a second pair was left as a non-enriched (NE) control. At 8 months, more than half the subjects were killed for pelts, and 302 new females were recruited (half enriched: ‘late E’). Several signs of improved welfare or productivity emerged. Access to enrichment increased play in juveniles. E mink were calmer (less aggressive in temperament tests; quieter when handled; less fearful, if male), and less likely to fur-chew, although other stereotypic behaviours were not reduced. On one farm, E females had lower cortisol (inferred from faecal metabolites). E males tended to copulate for longer. E females also weaned more offspring: about 10% more juveniles per E female, primarily caused by reduced rates of barrenness (‘late E’ females also giving birth to bigger litters on one farm), effects that our data cautiously suggest were partly mediated by reduced inactivity and changes in temperament. Pelt quality seemed unaffected, but E animals had cleaner cages. In a subsidiary side-study using 368 mink of a second colour-type (‘Demis’), similar temperament effects emerged, and while E did not reduce fur-chewing or improve reproductive success in this colour-type, E animals were judged to have better pelts. Overall, simple enrichments were thus beneficial. These findings should encourage welfare improvements on fur farms (which house 60-70 million mink *p.a.*) and in breeding centres where endangered mustelids (e.g. black-footed ferrets) often reproduce poorly. They should also stimulate future research into more effective practical enrichments.

## Introduction

Environmental enrichment is the addition of physical or social stimuli to animals’ captive environments to improve their welfare e.g. [1]–[3], a definition reflecting its current usage in applied animal research and management, as opposed to its original use in neuroscience as a means of enhancing brain development [4], [5]. The stimuli provided typically allow or encourage behaviour patterns that the animals are naturally motivated to perform (see e.g. [6]), examples including substrates in which pigs can root [7] and giraffe feeders that require tongue manipulation [8]. Many studies across diverse species, both wild and domesticated, demonstrate the success of enrichment for improving wellbeing: animals are often highly motivated to access enrichment items (e.g. [3], [9], [10]), and enrichment often reduces a wide range of signs of poor welfare, including stereotypic behaviours such as repetitive pacing or self-plucking (e.g. [11]–[13]), behavioural or physiological signs of fear (e.g. [3], [14]–[16]), and depression-like symptoms (e.g. [17]). In addition, perhaps because of its stress-reducing effects, environmental enrichment can enhance play (e.g. [18]–[20]), and even extend animals’ lifespans (e.g. [21]).

Growing evidence also indicates that enrichment can improve reproduction and have other practical advantages. Enriched-raised males copulate more often than non-enriched in fruit flies [22], [23] and American mink [24], while some female mammals show improved maternal care if enriched (e.g. increased pup-licking by rats: [25]; reduced piglet-crushing by sows: [26]). Furthermore, in some domesticated animals, enrichment may increase reproductive output: it can extend lifelong fertility in broiler breeder hens [27], and boost reproductive rates in laboratory mice [28], [29]. These reproductive benefits can increase producer profits, generating more income than the enrichments cost [27], [29]. Other reported practical benefits include increasing adoption rates at cat shelters [30]; accelerating post-operative recovery in research animals [31]; reducing biting by pet ferrets [20]); reducing feed consumption and the wastage of nesting material on mink farms [32]; and improving post-release survival in animals reintroduced to the wild from conservation breeding centres [33].

Our aims were to investigate the effects of simple, practical environmental enrichments on fur-farmed mink (*Neovison vison*). For those unfamiliar with mink farming, animals are housed in rows of hundreds of wire mesh cages, each with a nest-box, typically in open-sided sheds, and fed a meat-based paste at least once per day. Litters of altricial infants (‘kits’) are born in late April or early May. Litters are separated from their mothers about two months later (in North America, generally at approximately 6 weeks of age), and housed in sibling pairs at around 10 weeks. Juveniles continue gaining weight up to about 7 months of age, during which time their winter coat emerges. Most mink are then killed for their pelts (‘pelted’) in early December, typically using carbon monoxide. The subset chosen as breeding stock are housed individually, and then mated in February or March when they come into season. Between pelting time and mating, animals are food-restricted to lose excess body-fat; for this reason, stereotypic pacing and related activities (henceforth ‘locomotor stereotypies’) are most evident in January and February. Pregnant and lactating females are fed *ad libitum*. In North America, they are moved just before birth to slightly larger ‘whelping pens’; over the entire annual cycle, they are typically re-caged four to six times. Their individual identities are known from cards positioned above each cage, and moved with the animals.

Mink are very worthwhile subjects for two reasons. First, 50 million are reared and killed for pelts each year [34], [35], representing a peak global population of 60–70 million animals. More than half are farmed in countries such as China, Poland, and the U.S. where enrichments are not mandatory. Second, this species is a convenient model for wild carnivores housed in zoos and conservation breeding centres. Mink show Carnivora-typical locomotor stereotypies [36], and are closely related to several endangered mustelids (e.g. black footed ferrets and European mink) that are too rare and housed in too small numbers to be suitable experimental subjects themselves. Furthermore, some of these endangered wild mustelids in breeding centres are kept in quite small, non-enriched cages comparable to those on farms [37]–[39], and commonly have reproductive problems (e.g. reviewed by [24]).

Several previous studies on mink have investigated the effects of specially-built single enrichments such as water baths and running wheels, or large, structurally complex, diversely enriched cages (e.g. [9], [10], [24], [40]–[42]). These have shown that mink are highly motivated to access certain enriched environments, and that these can boost male copulation rates, and reduce physiological signs of stress along with locomotor stereotypies. However, such elaborate enrichment is not feasible on commercial farms, involving items that are costly to build or maintain, or need frequent replacement. Growing evidence suggests that simpler, more practical enrichments appropriate for small cages can also improve mink welfare, as indicated by measures of motivation, and decreased stereotypic behaviour and cortisol metabolite output. These include manipulable objects such as balls, structural additions to the cage like suspended wire mesh and shelves, and even ‘chunky’ food requiring more manipulation and time to consume than the minks' typical feed [32], [43]–[46]. However, their welfare effects are not always consistent, suggesting that simple enrichments of different types vary in efficacy, and that only providing enrichments once mink have reached adulthood may be ineffective [47]. To illustrate, some enrichments reduced one stereotypic behaviour, fur-chewing, but not locomotor stereotypies [46]; others even increased locomotor stereotypy, perhaps due to increasing general activity [44]; while others failed to reduce — or even enhanced — glucocorticoid output (e.g. [45], [46]). Furthermore, no studies have investigated whether any mink enrichments affect reproductive output, ease of handling or pelt price.

We therefore investigated how a practical enrichment program on North American farms affected mink welfare over the course of their annual cycle. To do so, we used a variety of measures indicative of welfare, predicting increased play, decreased fear and screaming, and reductions in stereotypic behaviour and cortisol levels that would not just reflect reduced general activity levels. We also assessed several measures that are potentially sensitive to welfare, but also important practically: male mating success and female fecundity, which were predicted to increase, and aggression, predicted to decrease. To further evaluate practical costs and benefits to farmers, effects on cage cleanliness, food consumption and pelt value were measured. Wherever possible, we also sought to assess how these effects inter-related, to try to infer possible mechanisms. The project began with a pilot study on two farms, followed by a more comprehensive experiment expanded to three farms. While not the largest enrichment experiment ever conducted (see [27] on broiler breeders), as far as we are aware, this is the largest-scale enrichment study ever conducted on a mammal, involving 18 months' data collection and c. 3200 animals.

## Methods

### Ethics statement

This work was approved by the University of Guelph Animal Care Committee, under protocol 10R108 (Animal Use Protocol. No. 1653).

### Experiment 1

The primary aim of this pilot study was to assess the practicality and cost of a wide range of possible enrichments, in order to choose a subset for a larger trial (Experiment 2). A subsidiary aim was to look for preliminary evidence of effects on welfare and productivity. This pilot focussed on the 5-month long period between the pair housing of juveniles and pelting time.

#### Subjects and housing

The experimental animals were 756 kits from 189 families across two farms in southern Ontario. They represented three strains bred for different pelt colours (colour-types): Blacks at Farm A, plus Demis (Wild-types/Browns in Europe) and Pastels at Farm B (farm nomenclature following that used in [45]). In July, kits were split into pairs and re-caged when approximately 10 weeks old. At this time, two sibling pairs were chosen from each family, one being randomly assigned to enriched (E) housing, the other to non-enriched (NE). These pairs were all male-female at Farm B, but a mix of male-female, male-male and female-female at Farm A (in different sheds across the farm). As part of Farm A's standard practices, 275 (120 female, 155 male) mink were then single-housed from September onwards, while the remainder were left in pairs until pelting. Farm B's animals all remained in pairs. Cages varied slightly in size across farms and different sheds, but were always 61 cm L x 23 cm W x 46 cm H or greater with an elevated wooden nest-box inside the cage.

E pairs received two or three of the enrichments listed in Table 1 in different combinations. Over the five months these were renewed if previous items were destroyed, lost from the cage or so soiled that they had to be removed, up to a maximum rate of once a month. Lost/soiled enrichments were typically replaced with similar items. The cumulative cost (in dollars) of enriching each cage over this period was recorded.

**Table 1:** Enrichment items.

Category	Specific items	Farmers' evaluation
Balls	*Golf balls*	Good; used by mink
	*Perforated plastic ‘wiffle’ balls*	Good as long as robust (manufacturers vary); used by mink
	Cat toys with bells	Too destructible and costly
Animal products	Pigs' ears, cows' ears, hide strips, cow hooves	Very attractive to mink, but too destructible
	Pieces of marrow bone	Too costly
Other chewing items	Cut portions of fire hose	Uninteresting to mink
	Plastic T-shaped plumbing fixtures	Good; used by mink
	Hanging lengths of garden hose suspended from the top of each cage	Good if tied securely
	Pieces of wood, wooden spoons	Uninteresting to mink
	Nylon rope, sisal twine	Too destructible; may unravel to create choking hazards
Tunnels	Large plastic pipes, wire mesh tunnels	OK if large/strong enough

*Italics indicate items that were selected for use in Experiment 2.*

#### Behavioural effects

Behavioural data were collected in November at Farm A. Live scans were conducted in the mornings in mid-November to assess enrichment use (as defined in Table 2). For these observations, only mink housed singly or in male-female pairs were used, to allow the reliable identification of individuals. In the same period, temperament was also assessed for these subjects using ‘stick tests’, in which a stick is partially extended into the cage. Mink were then categorized, based on their immediate reactions, as fearful (i.e. retreats or remains at back of cage attending to stimulus), curious (approaches and makes contact), aggressive (bites in a hard, sustained manner), or unresponsive (does not respond in any of the above ways within 30 seconds) [48]. This was to assess whether enrichments cause changes in fear or aggression as observed in other species (see Introduction); fear in these tests is also commonly used to assess mink welfare (e.g. [49]–[51]). The tests were conducted in the mornings prior to the behavioural scans; each cage was tested twice on consecutive days resulting in two scores per animal (four scores per cage for pair-housed mink).

**Table 2:** Ethogram for all behavioural observations.

Activity	Description
Stereotypic behaviour^1^	
Locomotor stereotypy	Movement or sequence of whole body movements repeated at least three times consecutively (‘scrabbling’ excluded; see below)
Borderline stereotypy	Apparent locomotor stereotypy interrupted before three repetitions or switching between elements of common stereotypies without repeating a sequence three times
Scrabbling	Repetitive scratching at wall of cage or nest-box
Wire-gnawing	Standing or lying with the mouth closed around the wire front of the cage
Inactivity	Lying still
Social play^2*^	Rough and tumble play (biting, sparring with paws, chasing, jumping onto cagemate), differentiated from aggression by the absence of hissing, screaming and/or persistent attempts to escape
General activity	Animal neither inactive nor engaged in stereotypic behaviour; includes eating, drinking and grooming, and enrichment use
E use	Mink is interacting with an enrichment while active; includes sniffing, carrying, moving or chewing it (thus excludes e.g. sleeping with an enrichment in the nest-box)
Object play^2^	A subset of E use excluding sniffing and similar exploratory behaviour: biting, pushing (with paws or nose), lifting, jumping on, manipulating (with paws), or chasing object

1 Assessment required interruption of scan with a focal observation of up to 10 s (cf. e.g. [10]).

2 Assessed in juvenile mink only, Experiment 2.

In late November, responses to handling were also assessed on both farms. All mink were removed from their cages and manipulated briefly by farm personnel to grade their pelt quality; this involved placing them under a light to inspect the pelt. Whether each mink screamed or squeaked in response was recorded. Squeaks are high-pitched, ‘breathy’, sometimes quiet vocalisations, often repeated in a bout, made in response to pain or fear [52]. Screams are louder and more prolonged, made in response to diverse threats, e.g. during fighting as well as when handled [24], [52], and made more often by fearful animals [53]. The workers handling the animals were blind to the hypothesis being tested, while the grader was typically blind to treatment (having no way to identify the animal's treatment during grading but possibly occasionally overhearing handlers stating an animal's identity).

Approximately a week later, the subset of mink selected for pelting (N = 391 NE, 374 E mink) were killed with carbon monoxide. Mink are killed in batches and so at this time individual identities and the ability to match siblings was lost; however E mink were killed in all-E batches, and NE mink in all-NE batches. Once killed, fur-chewing was assessed by scoring the bodies and tails of all of these mink for missing fur. The missing fur was often very slight (e.g. 0.5 cm), and was scored as a simple yes/no, with no attempt to assess severity.

#### Consequences for farmers: pelt quality and cage cleanliness

Effects of enrichment on pelt quality were assessed in several different ways. At Farm A, we recorded whether each mink was kept on as breeding stock (indicating both a good, large pelt as well as having a mother who had reproduced well) or pelted. For individuals that were pelted (N = 302), pelts were tagged and their bar codes recorded, so that the price for which they sold at auction could be assessed. At Farm B, when the farmer graded the pelt quality of live mink, these grades were recorded.

Cages where the mink had been housed were also scored after pelting, when empty (and after surviving animals had been re-caged, and enrichments removed), as clean or dirty, based on the presence or absence of accumulated faeces on the floor. This was done by an observer who was blind to the previous treatments (score 0 =  clean, 1 =  fur and dirt or faeces accumulated on wire).

At the end of the experiment, we selected specific enrichments for Experiment 2 based on apparent usage by mink, and on farmers' preferences, costs, and other practical considerations.

#### Statistical analyses

All statistical analyses were conducted using JMP 9 (SAS Institute), with the exception of binomial tests, which used the online calculator offered by GraphPad. Treatment effects on temperament and pelt grade were assessed using binomial tests [54] for differences between enriched and non-enriched siblings; for temperament, fear counts and aggression counts were totalled per cage (4 being the maximum), and for these variables, the counts from each E cage were compared to the NE cage containing its siblings. Screaming at grading and cage cleanliness were compared between treatments using chi square tests, both farms pooled. Pelt prices were compared using t-tests, after testing data to ensure that they met the necessary assumptions, and enrichment use was compared between housing types (pair or single) using t-tests for unequal variances. Fur-chewing proportions were compared using Fisher's Exact tests (due to small sample sizes). All tests were two-tailed. Results were considered significant at α<0.05, and trends are reported with p<0.10 throughout the paper.

### Experiment 2

#### Subjects and housing

Experiment 2 added a new site, bringing the number to three (Farm C being the same as in [45]). As well as enlarging the sample size, the aim of Experiment 2 was to use better standardised subjects than in Experiment 1, and to collect more detailed data, including data on reproductive variables. The initial subjects were 2400 mink kits from 600 families. Again, two sibling pairs were used from each family (now always male-female pairs), with one pair enriched when the kits were split into pairs in July, while the control pair was housed in standard conditions (non-enriched or NE). All were now kept in pairs until pelting. The aim had been to focus on Blacks, and thus all subjects were Black at Farms A and C; on Farm B, however, the farmer was interested in Demis and thus a mixture of Blacks and Demis were used (see Table 3 for details). For logistic reasons some data could not be collected for this second colour-type, and including them in the same analyses as the others might have obscured farm effects that we wanted to investigate; the Demis were thus treated as a side-study and data for them analysed and reported separately.

**Table 3:** Sample sizes per farm in each period in Experiment 2.

Farm, colour & sex	Autumn^1^ (growing period)		Pelted (December)		Winter/spring (mating through lactation)		
	E	NE	E	NE	Life E	Late E	NE
A – Black females	200 (69)	200 (69)	108	108	71	109	173
- with bunks^2^					16	19	39
– Black males	200 (69)	200 (69)	99	123	36	0	35
B – Demi/wild-type females	92 (30)	92 (30)	65	65	9	0	4
- Demi/wild-type males	92 (30)	92 (30)	74	75	5	0	7
B – Black females	108 (47)	108(49)	50	48	43	19	61
- with bunks^2^					6	8	9
– Black males	108 (47)	108 (49)	56	65	15	0	15
C – Black females	200 (100)	200 (100)	54	60	126	30	145
- with bunks^2^					23	10	25
– Black males	200 (100)	200 (100)	151	148	32	0	39
**Total**	**1200**	**1200**	**657**	**692**	**337**	**158**	**479**

N.B. numbers pelted and present in winter/spring do not sum to autumn totals due to loss of identity cards during moves, as discussed in the text.

1 Numbers in brackets indicate sample sizes for behavioural data.

2 These individuals are the subset of the total number of females listed in the row above which were given bunks.

**Table 4:** Summary of enrichment effects on known or likely welfare indicators in Black mink in Experiment 2 (see also Figs. 3 & 4).

	Effect	E^1^	NE^1^	Statistics
Play (% of observations; Farm A only)	**Increased**	7.0	5.6	F_1,97_ = 58.09, p<0.001
Screaming when handled	**Decreased** (where siblings differed)	89 of differing siblings	119 of differing siblings	Binomial test p = 0.044
Fear – juvenile	NSD	73 more fearful than sibling	56 more fearful than sibling	Binomial test p>0.10
- adult (Farms B & C)	**Decreased** Males only			Sex*treatment χ^2^ = 4.12, p = 0.042
	Males	11% of 44 mink	28% of 54 mink	?^2^ = 4.84, p = 0.028
Aggression - juvenile	**Decreased** (where siblings differed): Farm C only	8 more aggressive than sibling	20 more aggressive than sibling	Farm C: Binomial test p = 0.036
- adult (Farms B & C)	**Decreased**	1% of 143	7% of 146	?^2^ = 7.94, p = 0.005
Total stereotypic behaviour (percent of scans)	NSD	14.7 (12.8–16.7)^2^	12.5 (11.0–14.3)	p>0.10
Siblings only	***Increased***	15.3 (12.3–18.9)^2^	11.7 (9.4–14.4)	p = 0.0677
Locomotor stereotypy – prevalence	NSD	90.9% of 110 mink	81.0% of 116 mink	Logistic regression p>0.10
- proportion of activity in stereotypers only	NSD	19.7% (16.8–23.0)^2^	19.7% (16.8–23.0)	Farm*sex*treatment F_2,395_ = 3.26, p = 0.028(logit-transformed); no treatment effect within groups
Fur-chewing	All: NSD	7.3% of 573 mink	10.1% of 566 mink	?^2^ = 2.59, p = 0.108
	**Relatively severe: Decreased**	0.9% of 573 mink	2.5% of 566 mink	?^2^ = 0.449, p = 0.035
Faecal cortisol metabolites (females; ng/g)	**Decreased** Farm A only	66.7 (49.9–90.0)^3^^,^ 4	108.9 (75.9–154.5)	Treatment*farm log-transformed F_2,142_ = 3.58, p = 0.031; Farm A: t = −2.13, p = 0.036
Faecal cortisol metabolites (males; ng/g)	NSD	103.8 (82.0–131.4)^4^	122.1 (95.9–155.4)	p>0.10

Italics indicate a statistical trend 0.05<P<0.10. NSD  =  no significant difference (p>0.10). Paired analyses were conducted for siblings for all adult behaviour; however, results are only presented where they differed from those in the larger group.

1 Means with standard deviations for continuous data unless otherwise specified; proportions for categorical data.

2 Back-transformed means with 95% confidence intervals from logit-transformed data.

3 Effect still present after controlling for overall activity levels (time spent doing anything other than resting; p>0.10).

4 Back-transformed means with 95% confidence intervals from log-transformed data.

**Table 3.** Sample sizes per farm in each period in Experiment 2.

**Figure 1:**
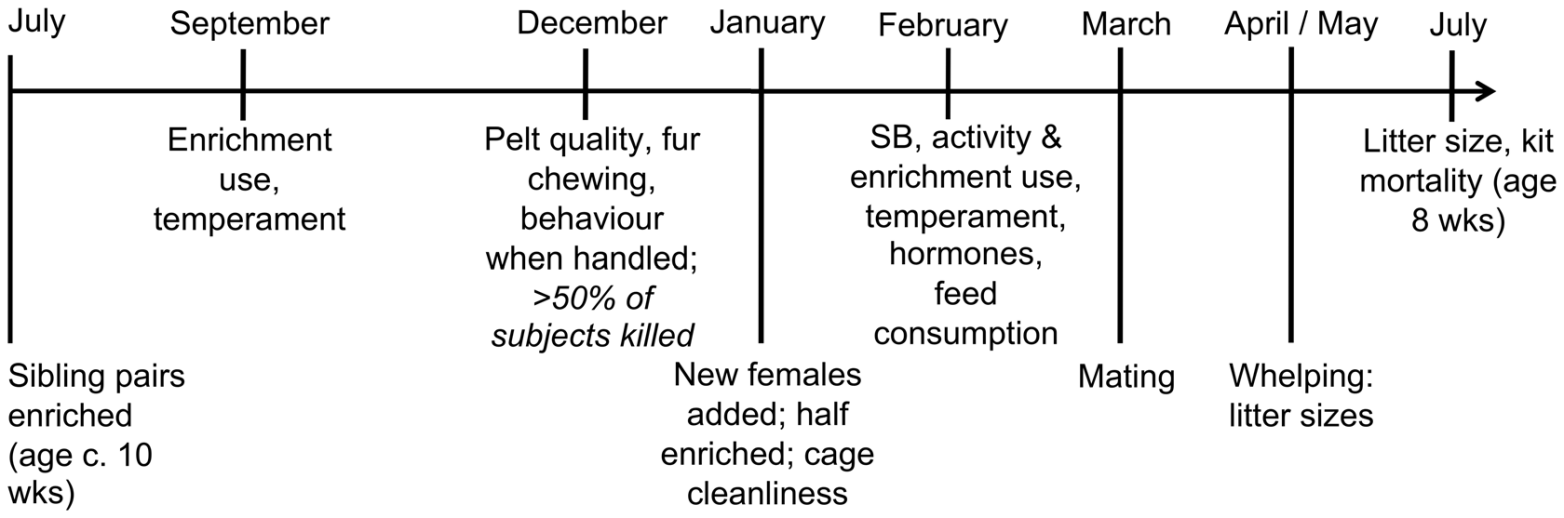
Simplified timeline of Experiment 2. SB  =  stereotypic behaviour.

Over half of these mink (and c.70% of males: see Table 3) were pelted in December, yielding 851 pelts from which to collect auction data, with the remaining mink being followed through the breeding season. To boost sample sizes for the reproductive phase, a supplementary group of 158 Black females of the same age was added in January (termed the ‘late E’ group, compared to the ‘life E’ group of original enriched subjects), and 144 non-enriched controls. Female reproductive data were thus collected from these new animals as well as the original subjects, while all other measures were recorded from original subjects only. A full timeline is provided in Figure 1.

All enriched animals were provided with three items inspired by Experiment 1 (and costing at most $4-6 per cage): a golf ball, a perforated plastic ball (practice golf balls or ‘wiffle’ balls), and a hanging object that could be chewed (chosen by each farmer), inspired by but not the same as those used in Experiment 1. At Farm A, this hanging object was a piece of garden hose (as in Experiment 1), one per cage; at Farm B, a length of plastic chain (a new enrichment); while at Farm C, half the enriched cages had hose and half had chain (alternating along the row of cages). To prevent these from being ejected from the cages (a problem in Experiment 1) and make them more interesting to the mink, these chains or hoses were hung from wires strung horizontally above the row of cages, such that tugging on one by a mink would move those in neighbouring cages — movement that we hoped would reduce habituation. These types of enrichment were sustained until just before mating, in males (see below), and until whelping for females kept for breeding. Pregnant females were moved before parturition to their whelping cages (61 cm×38 cm×46 cm or greater, with a nest-box attached to the front where straw or shavings were provided as bedding). Here, the hanging objects were simply suspended from the top of the individual cage (not from a horizontal wire). Furthermore, because balls and other objects were often carried into the nest-box in Experiment 1, no balls were provided from the time females were moved into these cages until their litters were approximately three weeks old, due to concerns for kit safety. A subset of females (details in Table 3) also received a structural enrichment to the cage: an elevated resting bunk installed when their kits were 21 to 24 days of age (see [45]). Over the course of the experiment, subjects were moved multiple times as part of normal farm practice. After each move, E animals were either given new enrichments, or recycled old enrichments that had been washed; no attempt was made to standardise these nor assess whether the two procedures had different effects.

#### Enrichment use and temperament (juveniles)

Behavioural data on enrichment use were collected on all three farms for a subset of 100 families per farm, between July and September, with more detailed behavioural data on play being collected on Farm A. These data were collected by instantaneous scan sampling conducted during daylight hours, for a total of 10 days per farm. This had to be done opportunistically with exact times varying between farms, because feeding time varied within farms and differed widely between farms at this time of year. Where feeding happened early in the day, observations ceased during feeding and for 20 minutes afterwards. The same 100 families from which these time-budget data were collected were also screened for temperament in the stick test in November, as in Experiment 1. The tests were conducted in the mornings, before feeding, and were repeated twice for each pair on separate days. On Farm B, these data were collected by an undergraduate who was blind to the hypothesis under test.

#### Measures at pelting time and beyond

In late November, animals were graded for size and fur quality by the farmers. As in Experiment 1, we recorded whether mink squeaked or screamed during this process; and as before, the workers handling the animals were blind to the hypothesis under test, while the grader was typically blind to treatment. Whether mink were selected to be kept for breeding or killed was also noted, as were live grade scores. In early December, when most of the mink were killed in batches for their pelts, pelts were grouped as ‘enriched’ or ‘non-enriched’, and scored for signs of fur-chewing as in Experiment 1 (with the refinement that any animals chewing more than 2 cm from the tips of their tails were noted). At Farms 2 and 3, pelts were then tagged to indicate these treatments and their bar codes recorded so as to follow them through auction.

After subjects were pelted, enrichments were removed from the cages they had occupied throughout the autumn. Cages were then scored for cleanliness by observers who were blind to the previous treatment, as in Experiment 1.

#### Behaviour and temperament (adult)

In February, when the remaining mink had reached sexual maturity, hose use was scored for all mink given that form of enrichment by inspection of the hoses' condition (0 =  no marks; 1 =  teeth marks; 2 =  hose torn; 3 =  hose destroyed completely). This was to validate behavioural observations with a metric reflecting longer-term, round-the-clock use: scans were not ideal for picking up the relatively rare, short duration bouts of enrichment use (equivalent assessments were not possible for the chains because they were made of hard plastic). Then, the behaviour of the same subset as in the autumn was observed again. Enrichment use was noted, but the primary purpose of these observations was to assess time spent performing stereotypic behaviour and their overall levels of activity (see Table 1). These observations were done by live scan sampling from 08:00 to 13:00 for 5 days per farm, using the ethogram in Table 2.

Stereotypic behaviour data were used to calculate two dependent variables. One pooled all forms previously shown to be reduced by enrichment, expressed as a proportion of observation time (e.g. [32], [55]): thus locomotor stereotypy plus scrabbling plus ‘borderline’ locomotor stereotypy (see Table 1). The occasional observations of wire-gnawing were excluded, since this appeared qualitatively different (not very repetitive in any bout) and had previously been shown not to be reduced by enrichment [32]. The second was locomotor stereotypy expressed as a proportion of total activity, since this is closely linked to perseveration (general tendencies to repeat behaviour in a functionless way: [55], [56]); it was also the only stereotypic behaviour metric reduced by a structural enrichment, elevated shelves for nursing dams, in an earlier study [57].

After this set of behavioural observations, these mink were also screened again for temperament. These data were only successfully obtained on Farms B and C. This time, a ‘glove test’ was used: a modified version of the stick test that uses a farmers' mink-catching glove in place of the stick. This increases the sensitivity of the test for detecting fear, making it more appropriate for adult mink in this population, where fear is relatively rare [58]. These tests occurred in the afternoons, after feeding.

#### Endocrine effects

Power tests suggested that a minimum of approximately 30 mink per group were needed to detect a housing effect of equal size to that found in a previous study [59]. Faecal samples were collected from at least that number of mink per farm and treatment, resulting in a total sample of 188 females across all farms, representing all sister pairs housed in the same randomly chosen area of one shed per farm. The samples were collected over a 24 h period in February, ending at approximately the time the winter behavioural observations began, and were used to assess faecal cortisol metabolites. Two weeks prior to mating, faecal samples were also collected from 70 males (all in one row in one shed) at Farm A to assay a broader range of steroid hormone metabolites: those of androgens as well as cortisol. All samples were frozen after collection. Steroids were then extracted by thawing and homogenising the samples and placing an aliquot in 80% methanol [60]. After shaking and centrifugation, extracts were analysed using an enzyme immunoassay [61] that has been validated for cortisol metabolites for this species [62]. Androgen metabolites were analysed with a testosterone and epiandrosterone enzyme immunoassay developed and validated for boars and previously applied to mink [24], [63].

#### Feed consumption

On Farms B and C, individual portions of feed for a total subset of 86 female mink (all sibling pairs, one E, one NE, in a row at a random location on each farm) were pre-weighed to give an equal, known amount to each individual. Any feed left uneaten was then weighed the following day as an indicator of feed consumed. This was repeated two or three times on each farm during the same period of February as the behavioural observations.

#### Reproductive behaviour (males) and success (females)

Behaviour at mating was observed for 54 males on Farm A (all previously sampled for faecal steroid metabolites). These males were moved to identical non-enriched cages, and given opportunities to mate with one to three females daily for a maximum of 17 days (females from the farm's main stock, rather than experimental animals). Females were placed in the male's cage until either copulation occurred, a lack of interest was established, or mink needed to be separated due to serious aggression. Two observers blind to male treatment collected data for the first mating of each day via scan sampling. The variables recorded were latency to ‘catch’ the female, latency to copulate; duration of copulation; and number of copulations obtained over the whole two-three week season. ‘Catching’ is the first phase of mating, in which the male bites the female's scruff and lies with her as she becomes quiet and receptive. Only times from successful mating attempts were used for analysis, along with the proportion of attempts in which the male successfully mated with the female. For males across all farms, the total number of successful mating attempts was also assessed using the farmers' records (counts on the animals' cards; unsuccessful attempts were not recorded but if several occurred the male stopped being used as a breeder).

Reproductive success in females was assessed via the proportion of females that produced litters (i.e. were not barren), the number of kits at the first count (done by the farmers within 1 to 2 days after birth; henceforth called ‘early litter size’), kit mortality, and consequent litter size at weaning (approximately 6 weeks after birth) including barren females.

#### Statistical analyses

Tests were run to investigate enrichment effects on our various welfare measures (play, fear, screaming, stereotypic behaviours, faecal cortisol metabolites); the measures both potentially sensitive to welfare and relevant practically (male mating success, female fecundity, and aggression); and on variables assessed solely for their importance to farmers (cage cleanliness; food consumption; pelt quality/price). On the one farm where both were supplied, we compared the efficacy of hoses and chains. In addition, whenever enrichment effects emerged in adult mink, we assessed whether they were predicted by observed enrichment use, and wherever possible we also assessed whether effects on reproductive benefits co-varied with effects on welfare indicators, to give some insights into potential mechanisms. Here, one-tailed tests were used as we had clear directional predictions: that usage and the various observed enrichment-effects should co-vary.

Analyses were conducted using JMP versions 10 & 11 (SAS Institute). Continuous data were analysed using general linear models (GLMs; [64]), while categorical data were analysed using logistic regressions or binomial tests (e.g. to assess whether siblings who differed, differed in one direction more than would be expected by chance). Interaction terms were removed from models if they had P-values greater than 0.25 [65]. In GLMs, normality and homogeneity of variance were assessed by inspection of the residuals, and transformations were applied where necessary. Locomotor stereotypy could not be made to meet the assumptions of these tests, and was therefore reanalysed in two ways: 1) recoded as whether absent or present, with treatment effects then analysed using logistic regressions; 2) a GLM to assess treatment effects on the time budgets of stereotypers only, i.e. with all non-stereotypers excluded (since this made residuals normalisable). Sequential tests were used in any GLMs that contained a continuous predictor variable likely to introduce non-orthogonality, with the term of interest (enrichment treatment) as the last main effect [64]. If transformations of continuous data were unsuccessful, non-parametric tests were used; thus pelt price data were analysed using Wilcoxon signed rank tests split by sex.

For all data collected before pelting, enriched animals were compared to their non-enriched siblings, for instance by blocking for family as a random effect (as well as blocking for farm, sex and their interactions, as fixed effects, where appropriate). After pelting, the ‘siblings as controls’ design broke down, because only one male and/or one female remained alive in many families. Data collected after pelting were therefore analysed in two ways. First, the data from all possible subjects were analysed with no control for family. However a new term specifying whether or not each individual had a known living sibling was added to each model (along with farm, sex, treatment and their interactions), since families in which both females and/or both males were kept for breeding were likely to be inherently higher in quality, and this factor had been included in similar analyses in other studies [45]. Second, data were then reanalysed for the subset of animals that did still have a living sibling; here, family was added to each model (as a random effect), along with farm, sex and their interactions. This ‘siblings’ dataset was much smaller and therefore potentially more prone to Type II errors than the full dataset; however the matched sibling design was inherently powerful, and where this enabled us to see effects that were otherwise undetected, these are reported.

For feed consumption, which was only analysed in sibling pairs, a matched pairs t-test was employed. For categorical data collected from live juveniles (temperament, screaming at handling), when all subjects were sibling pairs, binomial tests were used to compare treatments where siblings differed in their behaviour, and these were repeated for adult temperament in the subset of subjects with surviving same-sex siblings.

For female reproductive data, the full dataset of all possible subjects included the group who had been given enrichments late in life, in January when nine months old. In these models, treatment was therefore recoded as ‘Life E’ (for our original subjects), ‘Late E’ (for these more recently added subjects) and non-enriched, and contrasts were used where appropriate to test whether Life E and Late E together differed from NE. Models for weaning litter size analyses also controlled for whether the female had been given a bunk *post partum*. For litter size, early litter size included all individuals born even if they were then cross-fostered to other mothers. Barren females were excluded from early litter size analyses, and prevalence of barrenness was analysed separately in a nominal logistic regression controlling for farm. Weaning litter size, in contrast, counted only kits raised by that mother, regardless of their biological relationship to her (i.e. fosterlings were included), and barren individuals were included, so that weaning litter size analyses reflect the overall, farm level effect of all treatment influences on kit production. Analysis of male androgen levels was repeated controlling for levels of faecal cortisol metabolites in case of cross-reactivity of adrenal steroids in the assay, since a positive correlation between androgen and cortisol metabolites has previously been demonstrated [24].

## Results

### Experiment 1

#### Behavioural effects

Siblings tended to be similar in temperament in stick tests, with enriched and non-enriched pairs yielding equal counts of aggressive responses in 63 families, and equal counts of fearful responses in 53. However, in 66 families, enriched and non-enriched siblings differed in counts of aggression, and in 48 of these the enriched cage yielded lower counts. This effect was significant (binomial p = 0.0003). Enriched and non-enriched siblings did not, in contrast, differ in fearfulness (p>0.10). Across both farms, more non-enriched mink screamed when handled (12 of 391 [3.1%], compared to 5 of 374 [1.3%] enriched but this difference was not significant [p>0.10]).

Overall, mink used the enrichment during 5.5% of observations. Moveable enrichments were often found in the nest-box, apparently carried in there by mink.

Across both farms, there was a trend for more non-enriched mink to have chewed fur off their tails (4.6% of 391 vs. 2.1% of 374 enriched mink; Fisher's exact p = 0.072).

#### Pelt quality and cage cleanliness

Enrichment did not influence whether a mink was selected as breeding stock versus culled in December at Farm A (p>0.10). At live grading on Farm B, 49 of 200 [24.5%] enriched mink were graded as better than their non-enriched siblings, whereas 47 of 200 [23.5%] non-enriched mink were graded as better than their enriched siblings (p>0.10). Furthermore, amongst pelted individuals at Farm A, there was no significant effect of enrichment on pelt price (p>0.10). The proportion of cages that were dirty also did not differ between treatments (22% of 394 E cages vs. 20% of 396 NE cages, p>0.10).

#### Comparison of enrichment items' practicality

The costs of the enrichments over the whole period ranged from $0.75 per cage (wiffle and golf balls) to c. $10 per cage (marrow bone plus other enrichments), averaging $4.60. This price included replacing some items multiple times. In consultation with the farmers, we identified several enrichments as being impractical (e.g. too destructible or expensive) or apparently little used by the mink, based on farmers' and researchers' informal observations (see Table 1).

### Experiment 2: Results for Black Mink

#### Time budgets

In July to September (at 3 to 5 months old), enriched mink spent 2.4±1.0% of time using enrichments. In these juveniles, overall play was increased by 25% by the presence of manipulable objects (see Table 4), because levels of social play remained unchanged (Ahloy Dallaire et al. unpublished data), and object play added to this. By February, clear enrichment use was reduced from its earlier levels to 0.4±0.1% of observations in the life E group. Hose damage scores strongly co-varied with the hose use recorded in behavioural scans (logistic regression, χ^2^ = 17.7, p<0.0001).

Results for stereotypic behaviour are presented in detail in Table 4. Overall, the proportion of the time budget occupied by all stereotypic behaviours did not significantly differ between housing treatments, nor did the prevalence of locomotor stereotypy. Among those mink who performed locomotor stereotypies, active time budgets were affected by an interaction between farm, sex and treatment, but treatment effects within each group were not significant. Focussing on siblings only revealed a trend for E animals to spend more time on all stereotypic behaviours than their NE siblings. However, this could be a by-product of increased activity levels (see Introduction): enriched mink were indeed significantly less inactive (45.7±1.1% vs. 50.4±1.1% of observations; F_1,551_ = 10.0, p = 0.002). Furthermore, adding overall activity levels to the model as a control eliminated the apparent trend increase in stereotypic behaviour in E compared to NE siblings (see Table 5). Fur-chewing, the other stereotypic behaviour investigated, was reduced in severity by enrichment: the overall prevalence of fur loss did not differ between treatments, but fewer enriched mink had relatively severe (>2 cm of the tail tip) fur loss.

**Table 5:** Summary of enrichment effects on male reproduction and other productivity-related variables in Black mink in Experiment 2.

	Effect direction	Effect size	Statistics
**Male reproductive measures**			
Number of successful matings (all farms)	NSD	10 (7–14) vs, 19 (6–14)^1^	Z = −0.03, p>0.10
Farm A only: Percent of mating attempts successful	NSD	61.0±0.1 vs. 57.0±0.1	p>0.10
Time to “catch” female to copulate: all successful males (min)	NSD	16.1±0.8 vs. 16.0±0.8	p>0.10
Latency to copulate: all successful males (min)	NSD	28.4±1.5 vs. 29.0±2.7	p>0.10
Duration of copulation: all successful males (min)	***Increased***	47.8±3.7 vs. 40.2±2.1	Welch's t_1,41_ = 1.76, p = 0.085
Brother pairs^2^	**Increased**		Sign test M = −3.50, N = 9, p = 0.039
Testosterone (ng/g)	**Decreased**	26 (23–41.5) vs. 39 (27.5–50.5)^1^	z = 2.45, N = 70, p = 0.014
Epiandrosterone (ng/g)	**Decreased**	6.7 (5.3–9.3) vs. 9.0 (6.2–12.5)^1^	z = 1.96, N = 70, p = 0.049
**Other practical consequences (both sexes)**			
Feed left uneaten (g)	NSD	18.0±2.4 vs. 18.4±2.3	p>0.10
Pelt prices (USD)	NSD	Females: 80.4±0.8 vs. 80.5±0.9; Males: 118.2±0.8 vs. 117.1±0.9	p>0.10
Cage cleanliness	**Increased**	42.9% vs. 35.2% clean	?^2^ = 4.80, N = 799, p = 0.0284

Italics indicate a statistical trend 0.05<P<0.10. Only female reproductive measures include the late E group.

**Figure 2:**
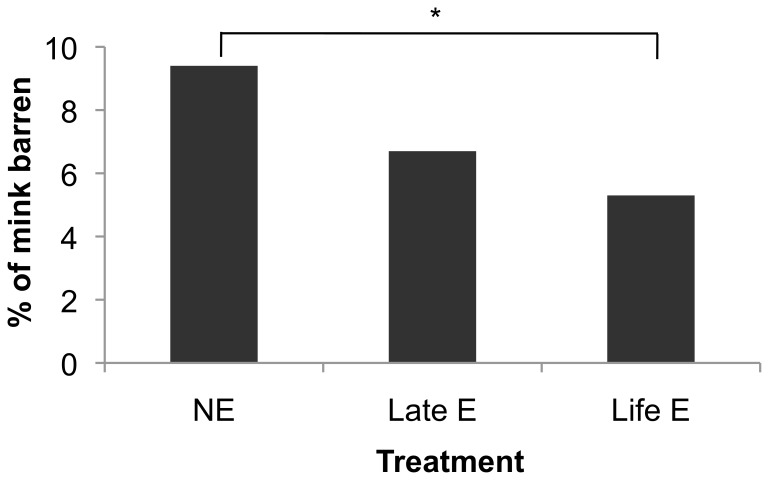
Rate of barrenness by treatment, Black female mink. * indicates the pairwise comparison that was statistically significant (NE vs. life E: p = 0.003, odds 2.62). The overall treatment effect was also significant (χ^2^ = 8.92, p = 0.012).

**Figure 3:**
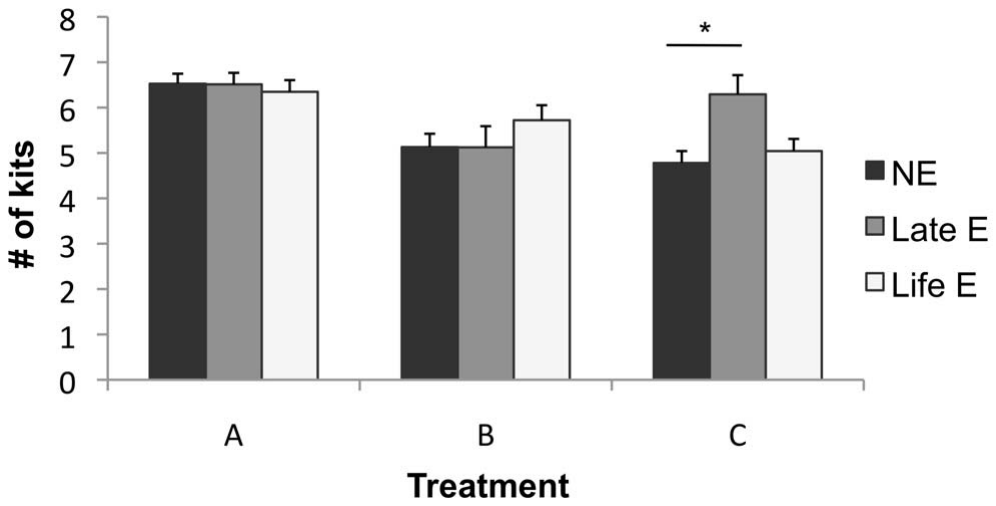
Early litter size of Black females who produced litters. Data are least squares means ± standard errors. Farm*treatment was significant: F_4,604_ = 3.56, p = 0.007. * indicates a significant treatment effect according to Tukey's HSD tests. Overall, enrichment for any length of time increased early litter size (contrast: F_1,604_ = 4.95, p = 0.026).

**Figure 4:**
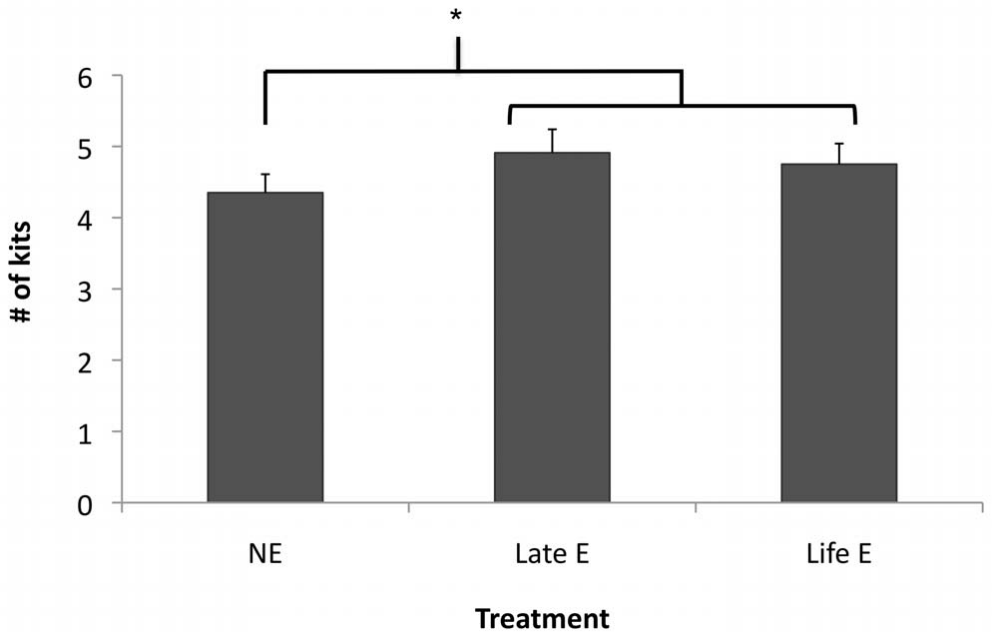
Litter size at weaning of all Black females. Data are least squares means ± standard errors. * indicates a significant difference at p<0.05. The overall treatment effect was only a trend (F_2,685_ = 2.74, p = 0.065); however, a contrast comparing the two enrichment treatments with non-enriched mink was statistically significant (F_1,685_ = 5.47, p = 0.020).

For effects on female reproduction, see Figures 2, 3, 4.

1 Medians with interquartile ranges in parentheses. These analyses do not control for cortisol metabolites levels, but doing so did not influence the outcomes.

2 Paired analyses were conducted for siblings on all measures of reproductive success; however, results are only presented if significant effects were detected that were not apparent in the larger group.

#### Temperament

Again, statistical details for all temperament analyses are presented in Table 4. In juvenile mink, aggression in stick tests was reduced by enrichment on one of the three farms. Fear in these tests was unaffected. However, where siblings differed, E mink were significantly less likely than their NE counterparts to scream when handled. Once the mink were mature, glove tests showed that enrichment now decreased aggression across all three farms, and decreased fear in males.

#### Pelt quality

Live grade scores did not differ between enriched and non-enriched siblings (p>0.10). Whether an animal was selected to be kept as a breeder rather than culled also did not differ between enriched and non-enriched siblings (p>0.10). Furthermore, prices fetched at auction did not differ between enriched and non-enriched mink pelts (p>0.10; see Table 5).

#### Feed consumption and cage cleanliness

Feed intake was not affected by environmental enrichment. When cages were scored simply as clean or dirty, enriched mink were more likely to have clean cages. Statistical details are presented in Table 5.

#### Endocrine parameters

Treatment interacted with farm to predict adult females' faecal cortisol metabolites. Transformations could not resolve problems with non-normality in these data, and non-parametric analyses were therefore used to assess differences within farms. These showed that at Farm A only, levels of faecal cortisol metabolites were lower in E than NE females (see Table 4). In males, cortisol metabolites did not significantly differ between treatments, but levels of both groups of androgen metabolites were unexpectedly lower in E males (see Table 5 for details).

#### Reproductive behaviour and success

Detailed results on male reproductive behaviour are presented in Table 5. In summary, enrichment did not influence the probability of males mating with the females presented to them, but did tend to increase the duration of their copulations when successful.

Among females, rates of barrenness were significantly reduced by life-long enrichment (for statistics and details, see Figure 2), but not by late enrichment. Early litter size for those who produced litters, meanwhile, was increased by the provision of enrichment objects when all E mink were contrasted with NE. However, this effect differed in strength between farms, the only significant effect being for late E at Farm C (see Figure 3). The proportion of kits born that died before weaning did not significantly differ between treatments (10.2±0.9% of litter vs. 11.1±0.9% of litter; p>0.10). The beneficial effects on barrenness and litter size at birth, not offset by any increases in kit mortality, thus caused a significant increase in the number of kits weaned by enriched mothers (see Figure 4). Unlike in a parallel study [45], however, bunks did not significantly reduce infant mortality (p>0.10).

#### Comparison of hoses and chains at Farm C

Mink tended to interact more with chains than hoses (0.2% of observations vs. 0.07% of observations; Welch's F = 3.59, d.f. 1, 96.6, p = 0.061). For total stereotypic behaviour as a proportion of observations, there was no effect of enrichment type (p>0.10). However, in males, enrichment type influenced the prevalence of locomotor stereotypy: this tended to be more likely to occur in those with chains than those with hoses (87.5% of 16 vs. 53.3% of 15, Fisher's exact p = 0.054). In females, in contrast, there was no difference (60.9% of 46 vs. 64.3% of 42, p>0.10). Furthermore, looking at locomotor stereotypy as a proportion of activity among mink who exhibited this behaviour, there was no effect of enrichment type in either sex (p>0.10). The effects of enrichment on adult temperament did, in contrast, depend on enrichment type: looking at sibling pairs who differed in fear or aggression, there was a trend for differential effects on fear. Of 11 pairs with hoses, the E sibling was less fearful than their NE sibling in only 3 (27.3%), but less fearful in 8 of 11 (72.7%) pairs with chains (Fisher's exact p = 0.086). For aggression, no differences could be detected because there were only four pairs where siblings differed on this farm. Life-long enrichment with hoses versus chains did not differentially influence litter size at weaning, the most comprehensive measure of female reproductive success (p>0.10). Similarly, males with chains and those with hoses did not differ in the number of times they were mated (p>0.10).

#### Correlations between enrichment effects

The following results are all one-tailed. Enrichment use in February correlated negatively with total stereotypic behaviour (F_1,553_ = 7.55, p = 0.003). There was no difference in the proportion of mink that exhibited locomotor stereotypy between those seen interacting with enrichments in February (N = 50) and those not seen interacting with them (73.0 vs. 68.0% of mink; p>0.10), and nor did the proportion of active time spent on locomotor stereotypy correlate with enrichment use in the same period. Enrichment use also did not predict barrenness, early litter size, nor litter size at weaning (P>0.10).

Relationships between reproductive success and temperament or inactivity could only be assessed for the subset of mink for which behavioural observations had been conducted, which did not include any late E individuals. Screaming during handling had to be excluded, although affected by treatment: its relationships with other enrichment benefits were not analysed because individual identity as used for the other measures could not always be reliably recorded during that procedure.

Across all males who mated, inactivity negatively correlated with copulation duration (F_1,46_ = 4.65, p = 0.018). Testosterone levels were not significantly positively correlated with copulation duration, nor was epiandrosterone (indeed relationships were negative, but deemed non-significant because of the one-tailed nature of the tests).

Females who were aggressive in the glove test had smaller early litter sizes (F_1,113_ = 3.30, p = 0.036). Aggression did not, however, significantly predict barrenness or litter size at weaning (p>0.10). Among siblings only, the pattern was slightly different: again, aggression did not significantly predict barrenness (p>0.10), nor did it predict early litter size within any treatment group (although there was a treatment by aggression interaction, F_1,72_ = 8.29, p = 0.008), but it was associated with smaller weaning litter sizes (F_1,83_ = 3.48, p = 0.033). Inactivity did not significantly predict barrenness in the full dataset or in siblings only. However, it interacted with farm to predict birth litter size in females who produced litters (F_2,273_ = 3.10, p = 0.047; at Farm C alone, inactivity predicted smaller litters: F_1,89_ = 3.85, p = 0.026). Results for siblings only were similar: inactivity was a significant predictor of smaller birth litter sizes (F_1,137_ = 4.70, p = 0.016). There was no significant relationship between inactivity and litter size at weaning in the full dataset (p>0.10). However, for siblings only, there was a significant negative correlation (F_1,142_ = 3.87, p = 0.026). Finally, when the correlates of faecal cortisol metabolites were investigated (only for mink from Farm A, where they were affected by enrichment), they did not significantly predict any measure of female reproductive success.

### Experiment 2: Results for Demi Mink

The responses of the Demis to enrichment were as follows (N.B. no data were collected on play, enrichment-use, pelt prices, faecal cortisol metabolites, male mating behaviour [aside from counts from cards], or feed consumption).

Where siblings differed in the stick test, enriched juveniles were less fearful in 41 of 60 cases (binomial p = 0.006). There were no significant differences in aggression (p>0.10). When handled, the Demis tended to follow the pattern seen in Blacks, E animals being less likely to scream than their NE siblings (binomial p = 0.09). In 77 of 119 pairs where siblings' pelts were graded differently during live grading, enriched animals received superior grades (binomial p = 0.036).

In adulthood, fear and aggression in glove tests did not differ between treatments (p>0.10). Effects of enrichment on stereotypic behaviour followed the same pattern as in Black mink. Total levels of locomotor stereotypy were increased by enrichment in siblings only (F_1,27_ = 9.01, p = 0.006), but this effect was not significant in the full dataset (p>0.10), and did not remain significant among sibling pairs after controlling for differences in activity (p>0.10). Furthermore, locomotor stereotypy as a proportion of active time did not differ between treatments in either dataset (p>0.10). Evidence of fur-chewing did not differ significantly between treatments (p>0.10).

In the full dataset, female Demis' reproductive success was not affected by enrichment according to any measure: rates of barrenness, early litter size, and litter size at weaning did not differ significantly between treatments (all p>0.10). The same was true when only sibling pairs were used in the analyses. Rates of barrenness were very low in the Demis, however, with not a single barren female among the females with living sisters. As in the Black mink, male mating counts were also not significantly different across treatments (p>0.10).

## Discussion

The items given to the Black mink in this study improved their welfare, as indicated by several measures. Increased play occurred during the juvenile period, suggesting potentially enhanced wellbeing [66], [67]: object play seems to be a rewarding activity, at least for young carnivores, since it is successfully used as a reinforcer [68]. The proportion of mink screaming when handled decreased, suggesting decreased fear [53], and males were less fearful in temperament tests (as were enriched Demi juveniles). Decreased fear obviously represents an improvement in welfare (see e.g. [48]). Our experimental mink also performed less severe fur-chewing in Experiment 2, supporting the trend for reduced fur-chewing seen in Experiment 1. Like other forms of stereotypic behaviour [69], those involving fur-plucking (or feather-plucking in birds) reflect poor welfare, being exacerbated by stressors during development (e.g. maternal deprivation) and current sub-optimal housing [70], [71], and in one mink study fur-chewing positively correlated with levels of cortisol metabolites [72]. This behaviour, typically directed at the tail, has therefore previously been used as an indicator of poor welfare or environmental disturbance for mink (e.g. [73], [74]) and is hypothesized to reflect stress and perhaps even boredom-like states in this species [72]. Furthermore, enrichment reduced faecal cortisol metabolites levels, albeit only on one farm. Baseline corticosteroid release in chronic conditions is far from a perfect welfare indicator because it can increase *or* decrease with poor well-being [75], [76] and activity levels are also a potential confound (e.g. [77]). However, in mink, long-term housing in highly preferred environments does reduce output of this hormone [24], [59] and controlling for overall activity levels did not affect the results here. Together, these findings show that even very simple cage additions can enhance mink welfare, and confirmed that the balls and hanging objects used were indeed environmental enrichments according to our definition.

Additional effects were also consistent with improved welfare, if not unequivocal proof. First, reduced aggression in temperament tests was displayed by both sexes and in both experiments. Aggressive reactions are harder to interpret than fearful ones. They could represent threat responses, suggesting that E mink found the test stimuli less threatening. Alternatively, they could represent predatory responses (see [58]), reductions then suggesting that predatory motivations are decreased in E animals, perhaps by interaction with enrichments. Enriched-raised females had improved reproductive success, primarily via reduced rates of infertility. Conception rates can be affected by stress, both directly via hormones of the hypothalamic-pituitary-gonadal axis (see [78]) and indirectly by decreasing attractiveness to potential mates (reviewed by [24]), although they can also reflect other variables such as levels of body fat [79], [80]. Our study did not aim to validate these variables as welfare indicators, and it is possible that effects we did not measure, such as increased body weight and/or reduced body fat, mediated some of these effects. Nevertheless, our preliminary finding that reduced aggression predicts better female reproduction cautiously suggests that beneficial psychological effects of enrichment were indeed important. Furthermore, regardless of welfare, these enrichment effects are highly beneficial for practical reasons.

Our finding of increased offspring production in enriched females is only the third study to show such benefits (adding to previous mouse and hen examples: see Introduction). It suggests that enrichment is both an effective way to improve mink productivity, and a useful tool for other mammals. Enriched-raised males also tended to perform longer copulations (important since copulation duration is linked to the resulting number of kits [81]). Previously there had been little scientific evidence that enrichment can improve mammalian reproduction [82], [83], despite anecdotal evidence for non-domesticated carnivores [83], [84]; reviewed by [24]. One reason for this effect in our subjects may have been the enriched minks' elevated activity levels. High inactivity has previously been linked to decreased litter size in mink and, less consistently, to increased risks of being barren [85]; cf. [79], [80]. Traditionally, farmers have enhanced activity and reduced body fat levels in breeding females via food restriction [86], [87]. Our new findings suggest enrichment provision as a supplementary or even alternative approach that is better for animal welfare [80]. Furthermore, for farmers, these reproductive improvements are financially advantageous. Averaging lifelong and late-enriched groups, enrichment objects resulted in an extra 0.48 kits weaned per female: an 11% increase. (Note that these effects are slightly different from those reported in [45], using a partially overlapping data set: this found that only late enrichment increased weaning litter size, but did so more markedly than in the present study, and across all farms. However, this study had used fewer subjects [45], and thus the current results are likely more accurate). With each pelt earning a profit of approximately $50 CAD, a 0.48 kit/female increase would yield an increase of approximately $24 per cage in return for at most $4–6 spent on enrichment (probably far less, as our prices did not involve bulk discounts). On a farm with 3000 breeding females, this would represent 1,440 extra pelts or a net profit of over $25,000 per year. This economic benefit was not offset by any detectable negative effects on pelt quality or feed consumption in enriched mink (and indeed was accompanied by an additional practical benefit: enhanced cage cleanliness). As a result, simple environmental enrichment of this kind, already required by law in Scandinavian countries, and recommended by a new industry Code in Canada [87] is likely to be willingly and widely adopted on mink farms everywhere.

Despite these successes, there were several limitations to this study, in terms of both methodology and enrichment effectiveness. The project's scale and geographical spread of the farms necessitated the use of multiple research assistants. While this allowed blinding [88], some assistants were inexperienced, and made errors despite being trained (cf. [89]; e.g. adult temperament data from one farm were judged unreliable due to discrepant scoring methods). Limited resources and personnel also meant that some potentially useful measures could not be collected, such as mortality rates (too hard to distinguish from subjects merely ‘lost’ during the frequent re-cagings); body weight and condition (data that would have been useful for understanding the reproductive effects on Blacks, and the improved live pelt grades of Demis); bite marks scored on the pelt leather (as evidence of agonistic interactions: [90]); certain in-cage behavioural data (e.g. time-budgets of late-enriched females); refusals to mate; and offspring growth rates. Furthermore, the welfare effects of our enrichments were not as marked as those of some mink studies. For example, cortisol metabolite reductions were inconsistent, and even when significant were much smaller than the c. 30% reductions seen in studies using more complex enrichments (cf. [24], [32]). As found in previous work on chewing ropes [46], our enrichments did not reduce locomotor stereotypies such as pacing and head-twirling cf. [24], [32], [91]; indeed these even increased in some cases (though this was mediated by increased activity, cf. [44], [92]). Our simple enrichments also did not enhance male mating rates, nor male androgen levels (instead paradoxically reducing these steroid levels) (cf. [24]). Our androgen results may reflect the timing of data collection: one study demonstrated that while fertile males had higher levels of testosterone than infertile males early in the winter, infertile males had the highest levels in February, when it was assessed in this experiment [93]. However, more research is needed to resolve this puzzle. Finally, levels of enrichment use by adults appeared low: only 0.4% in February, compared to levels around tenfold that in studies of adult mink with more complex, varied enrichments (4%: [10], 20%: [94]). Such limitations highlight the importance of continuing to search for practical enrichments that better enhance mink welfare, and illustrate how future research could improve on our methods.

Obvious directions for future applied research are thus to identify practical enrichment objects and regimes that better improve mustelid welfare and reproduction. In terms of object types, our main experiment suggested that plastic chains might be more effective than hoses (tending to be used more and better at reducing fear, despite paradoxical effects on one measure of locomotor stereotypy), but more detailed comparisons are needed for firmer conclusions. The pilot study also identified other enrichments with potential (plastic plumbing pieces, shelves/large tubes). Furthermore, informal observations during the pilot suggested that animal products were very attractive, although rendered impractical by price (bones from pet stores) or their tendencies to be rapidly destroyed (e.g. hide strips); thus, if low cost, robust animal products could be found, these too could have potential. In addition, other studies show that providing preferred resting places [10] or ‘chunkier’ food [46] are simple ways to reduce the locomotor types of stereotypic behaviour that our objects failed to address. As well as more formally comparing different objects and structures, future work should investigate whether reducing habituation could enhance the sustained use and impact of enrichments. For enrichments intended to elicit general manipulation and ‘play’ (rather than meeting specific behavioural needs), habituation is common [2], and in an earlier study of mink, this was clearly evident over a month's exposure to ‘play balls’ [44]. In the current work, habituation seemed likely (although not investigated directly): adults who had had enrichments for seven months showed very low interaction rates, while ‘late enrichments’ (added in January) boosted birth litter sizes on one farm. Future research should therefore see if rotating enrichment types (even moving objects between cages), or staggering their introduction (e.g. supplying a golf ball, wiffle ball and chew sequentially instead of simultaneously), would enhance effectiveness. Combining physical enrichments with other welfare-improving initiatives might also increase their efficacy [95].

Future applied studies should also investigate ‘farm effects’: how site differences in temperament, activity levels or husbandry influence the impact of enrichments. Our study suggests these are important, but in using only three sites, revealed little about what might drive them (with one possible exception: enrichment had no detectable effects on reproductive output on Demis or on the Blacks of Farm C, two sub-populations whose litter sizes were already high [around 6 kits] suggesting possible ceiling effects). No farms used throughout this study were positive for Aleutian disease. It would be interesting to assess enrichment effects on Aleutian-positive farms, where animals might be more vulnerable to opportunistic infections [96], since opportunistic infection rates are typically reduced by lowered stress (e.g. [97]). Relatedly, whether mink colour-types (strains) vary in what enrichments they prefer, and/or in how they respond to them, warrants further study. Colour-types with sub-optimal immune systems (e.g. Sapphires) could be especially interesting, as they too are more susceptible to disease [73], [98].

We hope our findings also inspire more research on other species, especially endangered mustelids like black-footed ferrets which are often kept in small, relatively unstimulating enclosures, and prone to reproductive problems [99], [100]. Finally, our work and similar recent studies (e.g. [46]) also raise fundamental new questions about environmental enrichment. First, why do enrichments differentially reduce different forms of stereotypic behaviour? Enrichments are thought to reduce stereotypic behaviours in three ways (e.g. [10]): by affecting brain regions important for behavioural sequencing and the control of ‘perseveration’ (inappropriate repetition); by satisfying specific frustrated motivations (e.g. to explore, hunt or range); and via ‘diversion’, reducing the time available to perform abnormal behaviour. Mink are ideal models for now investigating how such processes act since their diverse stereotypic behaviours (ranging from pacing to fur-chewing) likely vary in time budgets, underlying motivations, and the types and degrees of perseveration involved. Second, why do individuals vary in their utilisation of enrichment? Like other species [101], mink show consistent individual differences in enrichment use [10]). Perhaps surprisingly, active, stereotypic animals appear to interact least with enrichment objects [10]. This pattern could explain why in our study, high enrichment use predicted low stereotypy: this correlation may not reflect cause and effect, but instead that active stereotypic phenotypes are less motivated by manipulable items, for reasons as yet not understood. Third, how does enrichment affect female reproduction? There were hints that both increases in activity and reduced aggression were important. Whether these changes actually mediate improved reproduction, and if so, how, are interesting research questions. Finally, one of our least original, yet most puzzling, findings was that enrichment objects reduced threat responses during temperament tests and human handling. This resembles previous findings that enriched laboratory rodents and primates react more calmly to sudden sounds, human caretakers, and other potential challenges (see Introduction). How could simple inanimate objects have such generalised effects? We suggest that by reducing frustration or boredom (cf. [42]), enrichments create more positive ‘cognitive biases’ [102], such that all ambiguous stimuli become less likely to be perceived as threatening: an exciting hypothesis for future test.
